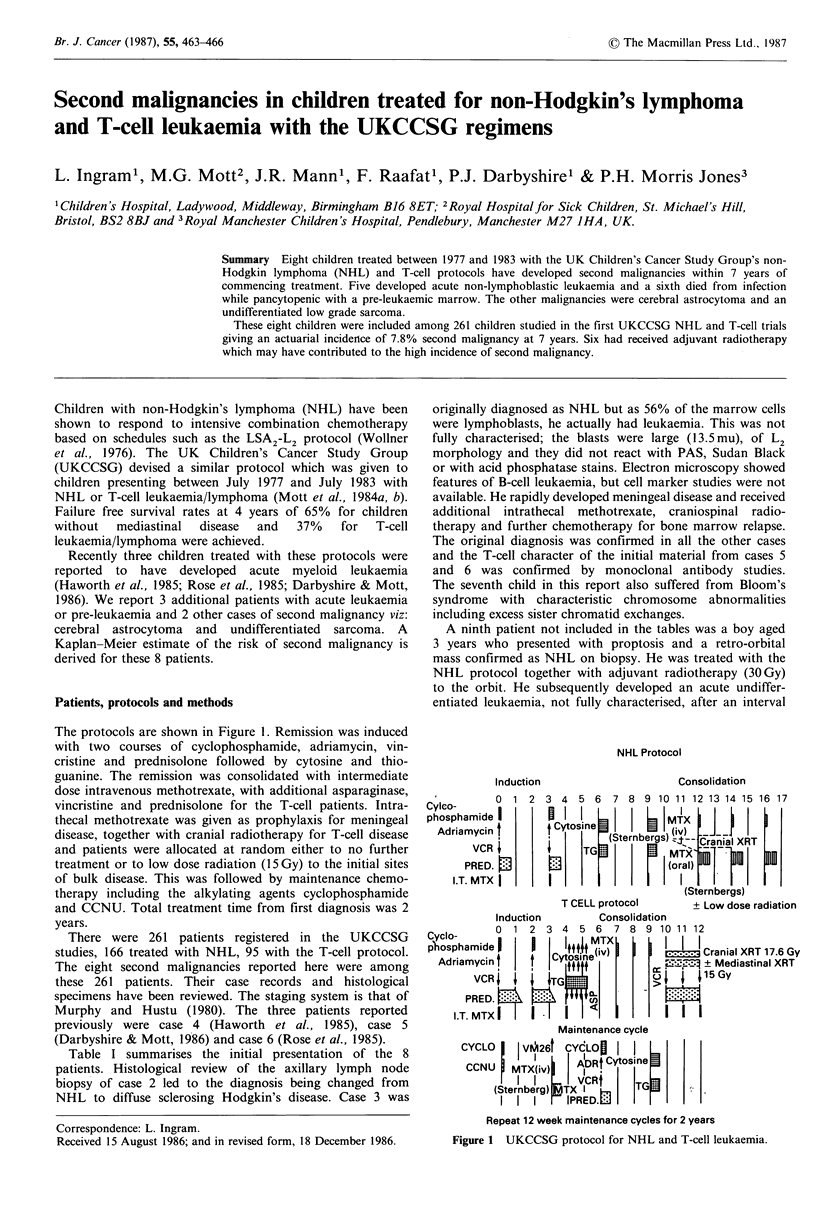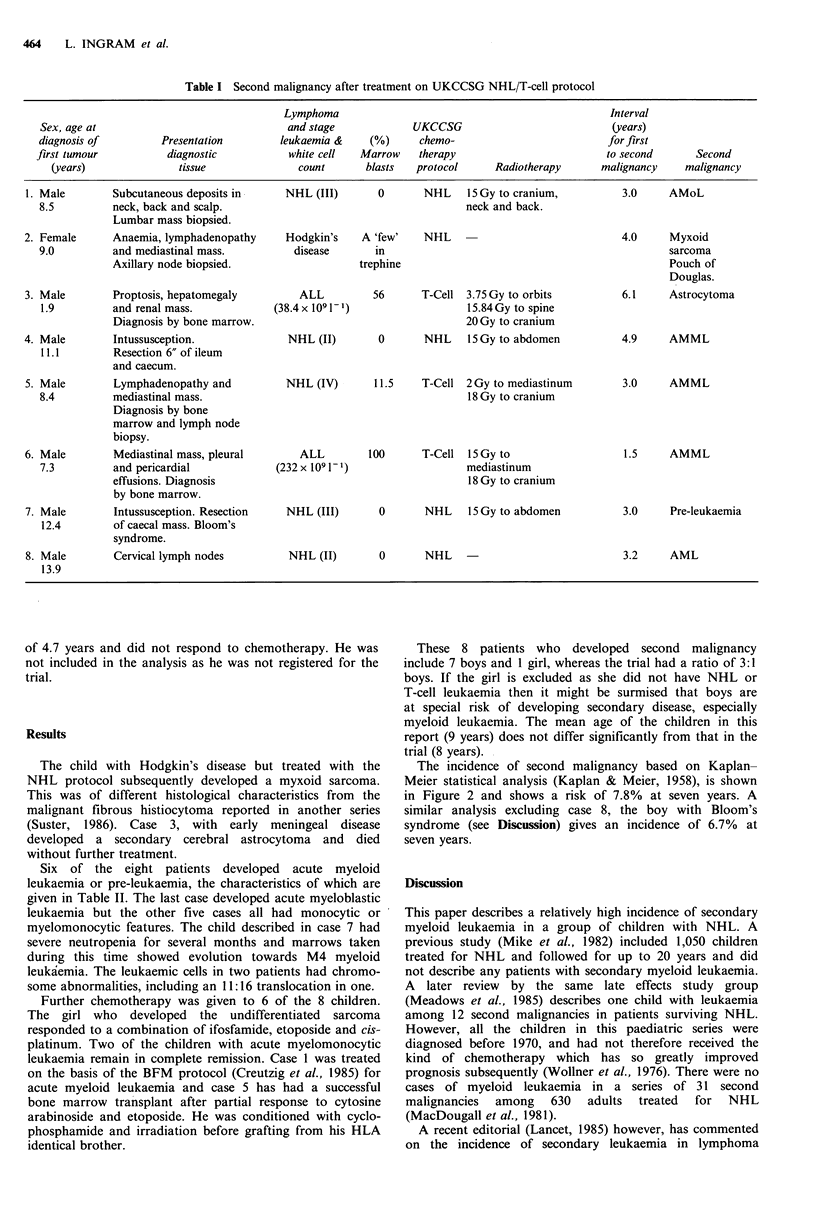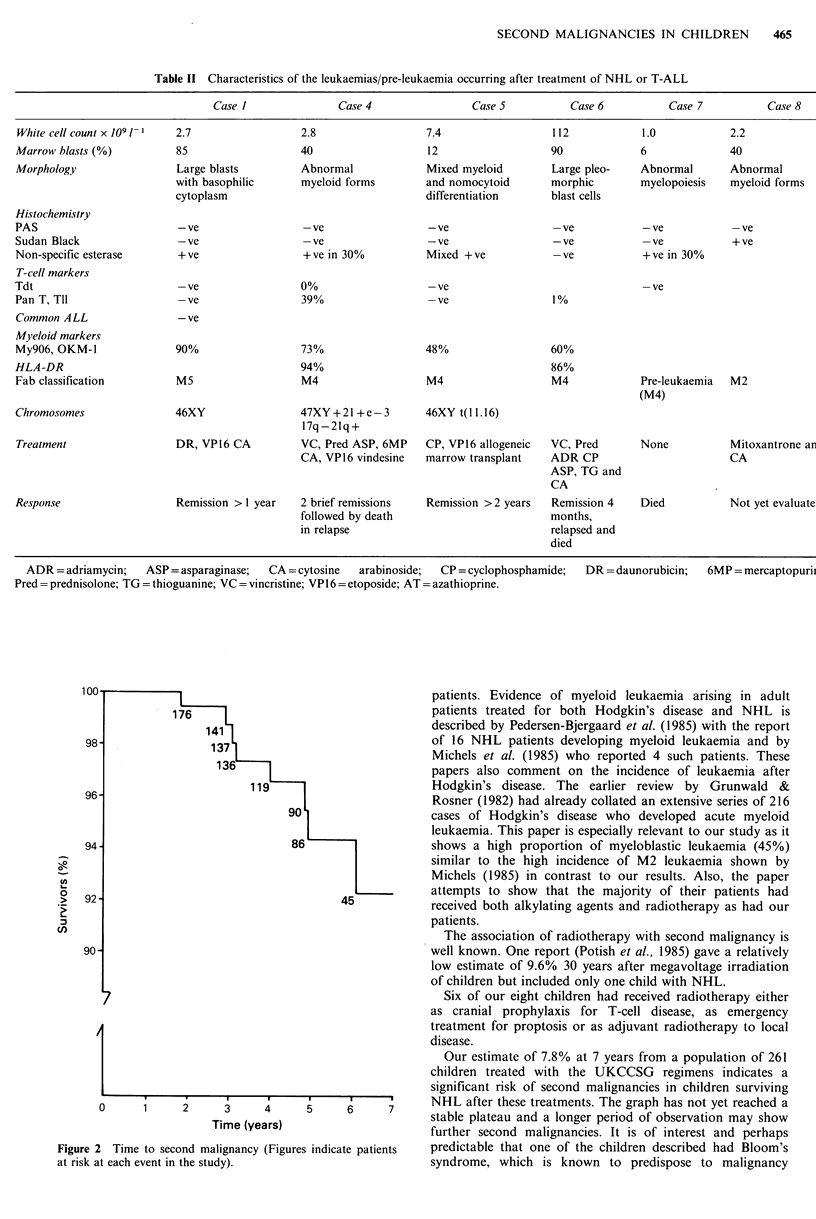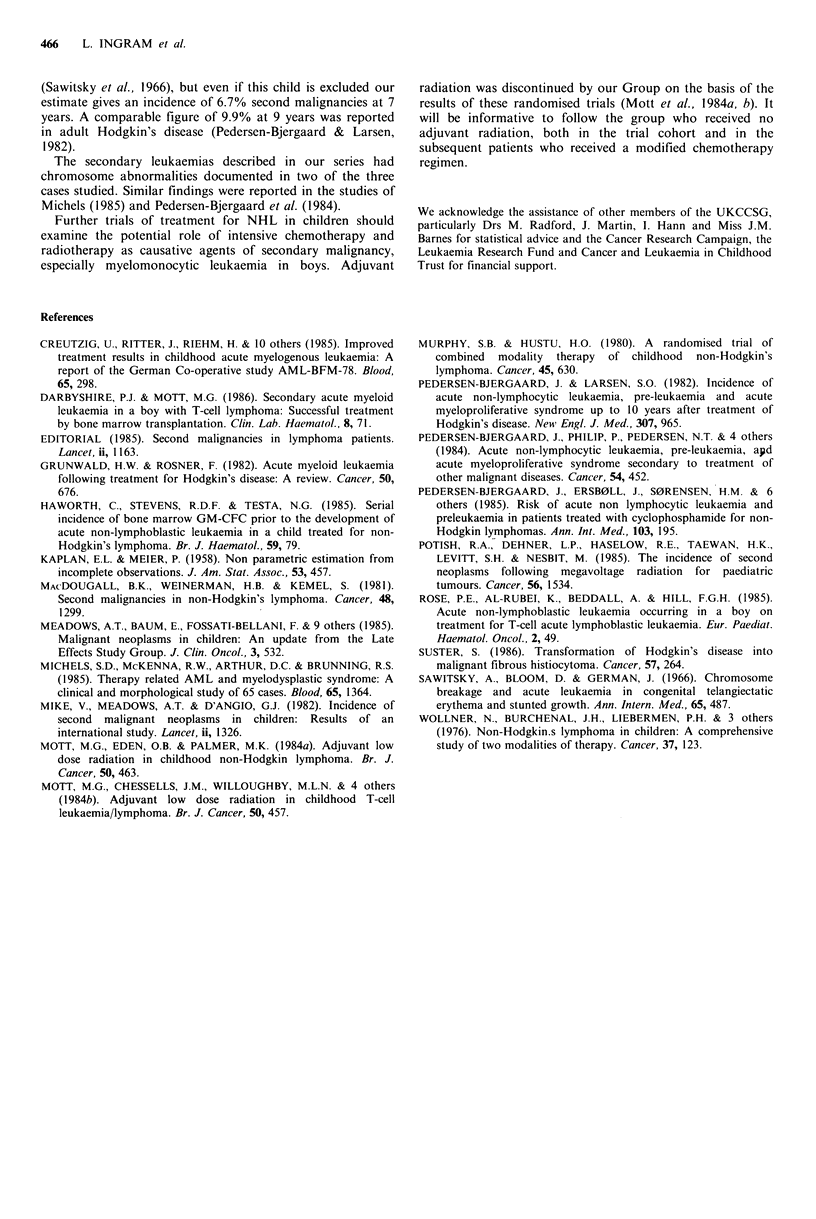# Second malignancies in children treated for non-Hodgkin's lymphoma and T-cell leukaemia with the UKCCSG regimens.

**DOI:** 10.1038/bjc.1987.91

**Published:** 1987-04

**Authors:** L. Ingram, M. G. Mott, J. R. Mann, F. Raafat, P. J. Darbyshire, P. H. Morris Jones

## Abstract

Eight children treated between 1977 and 1983 with the UK Children's Cancer Study Group's non-Hodgkin lymphoma (NHL) and T-cell protocols have developed second malignancies within 7 years of commencing treatment. Five developed acute non-lymphoblastic leukaemia and a sixth died from infection while pancytopenic with a pre-leukaemic marrow. The other malignancies were cerebral astrocytoma and an undifferentiated low grade sarcoma. These eight children were included among 261 children studied in the first UKCCSG NHL and T-cell trials giving an actuarial incidence of 7.8% second malignancy at 7 years. Six had received adjuvant radiotherapy which may have contributed to the high incidence of second malignancy.


*Br. J. Cancer* (1987), **55**, 463–466 © The Macmillan Press Ltd., 1987

### Second malignancies in children treated for non-Hodgkin's lymphoma and T-cell leukaemia with the UKCCSG regimens

L. Ingram[1], M.G. Mott[2], J.R. Mann[1], F. Raafat[1], P.J. Darbyshire[1] & P.H. Morris Jones[3]

[1]*Children's Hospital, Ladywood, Middleway, Birmingham B16 8ET;* [2]*Royal Hospital for Sick Children, St. Michael's Hill, Bristol, BS2 8BJ and* [3]*Royal Manchester Children's Hospital, Pendlebury, Manchester M27 1HA, UK.*

**Summary** Eight children treated between 1977 and 1983 with the UK Children's Cancer Study Group's non-Hodgkin lymphoma (NHL) and T-cell protocols have developed second malignancies within 7 years of commencing treatment. Five developed acute non-lymphoblastic leukaemia and a sixth died from infection while pancytopenic with a pre-leukaemic marrow. The other malignancies were cerebral astrocytoma and an undifferentiated low grade sarcoma.

These eight children were included among 261 children studied in the first UKCCSG NHL and T-cell trials giving an actuarial incidence of 7.8% second malignancy at 7 years. Six had received adjuvant radiotherapy which may have contributed to the high incidence of second malignancy.

Children with non-Hodgkin's lymphoma (NHL) have been shown to respond to intensive combination chemotherapy based on schedules such as the $LSA_2$-$L_2$ protocol (Wollner *et al.*, 1976). The UK Children's Cancer Study Group (UKCCSG) devised a similar protocol which was given to children presenting between July 1977 and July 1983 with NHL or T-cell leukaemia/lymphoma (Mott *et al.*, 1984*a*, *b*). Failure free survival rates at 4 years of 65% for children without mediastinal disease and 37% for T-cell leukaemia/lymphoma were achieved.

Recently three children treated with these protocols were reported to have developed acute myeloid leukaemia (Haworth *et al.*, 1985; Rose *et al.*, 1985; Darbyshire & Mott, 1986). We report 3 additional patients with acute leukaemia or pre-leukaemia and 2 other cases of second malignancy *viz*: cerebral astrocytoma and undifferentiated sarcoma. A Kaplan–Meier estimate of the risk of second malignancy is derived for these 8 patients.

### Patients, protocols and methods

The protocols are shown in Figure 1. Remission was induced with two courses of cyclophosphamide, adriamycin, vincristine and prednisolone followed by cytosine and thioguanine. The remission was consolidated with intermediate dose intravenous methotrexate, with additional asparaginase, vincristine and prednisolone for the T-cell patients. Intrathecal methotrexate was given as prophylaxis for meningeal disease, together with cranial radiotherapy for T-cell disease and patients were allocated at random either to no further treatment or to low dose radiation (15 Gy) to the initial sites of bulk disease. This was followed by maintenance chemotherapy including the alkylating agents cyclophosphamide and CCNU. Total treatment time from first diagnosis was 2 years.

There were 261 patients registered in the UKCCSG studies, 166 treated with NHL, 95 with the T-cell protocol. The eight second malignancies reported here were among these 261 patients. Their case records and histological specimens have been reviewed. The staging system is that of Murphy and Hustu (1980). The three patients reported previously were case 4 (Haworth *et al.*, 1985), case 5 (Darbyshire & Mott, 1986) and case 6 (Rose *et al.*, 1985).

Table I summarises the initial presentation of the 8 patients. Histological review of the axillary lymph node biopsy of case 2 led to the diagnosis being changed from NHL to diffuse sclerosing Hodgkin's disease. Case 3 was originally diagnosed as NHL but as 56% of the marrow cells were lymphoblasts, he actually had leukaemia. This was not fully characterised; the blasts were large (13.5 mu), of $L_2$ morphology and they did not react with PAS, Sudan Black or with acid phosphatase stains. Electron microscopy showed features of B-cell leukaemia, but cell marker studies were not available. He rapidly developed meningeal disease and received additional intrathecal methotrexate, craniospinal radiotherapy and further chemotherapy for bone marrow relapse. The original diagnosis was confirmed in all the other cases and the T-cell character of the initial material from cases 5 and 6 was confirmed by monoclonal antibody studies. The seventh child in this report also suffered from Bloom's syndrome with characteristic chromosome abnormalities including excess sister chromatid exchanges.

A ninth patient not included in the tables was a boy aged 3 years who presented with proptosis and a retro-orbital mass confirmed as NHL on biopsy. He was treated with the NHL protocol together with adjuvant radiotherapy (30 Gy) to the orbit. He subsequently developed an acute undifferentiated leukaemia, not fully characterised, after an interval

**Figure 1** UKCCSG protocol for NHL and T-cell leukaemia.

Correspondence: L. Ingram.
Received 15 August 1986; and in revised form, 18 December 1986.

**Table I** Second malignancy after treatment on UKCCSG NHL/T-cell protocol

| *Sex, age at diagnosis of first tumour (years)* | *Presentation diagnostic tissue* | *Lymphoma and stage leukaemia & white cell count* | *(%) Marrow blasts* | *UKCCSG chemo-therapy protocol* | *Radiotherapy* | *Interval (years) for first to second malignancy* | *Second malignancy* |
|---|---|---|---|---|---|---|---|
| 1. Male 8.5 | Subcutaneous deposits in neck, back and scalp. Lumbar mass biopsied. | NHL (III) | 0 | NHL | 15 Gy to cranium, neck and back. | 3.0 | AMoL |
| 2. Female 9.0 | Anaemia, lymphadenopathy and mediastinal mass. Axillary node biopsied. | Hodgkin's disease | A 'few' in trephine | NHL | — | 4.0 | Myxoid sarcoma Pouch of Douglas. |
| 3. Male 1.9 | Proptosis, hepatomegaly and renal mass. Diagnosis by bone marrow. | ALL ($38.4 \times 10^9 l^{-1}$) | 56 | T-Cell | 3.75 Gy to orbits 15.84 Gy to spine 20 Gy to cranium | 6.1 | Astrocytoma |
| 4. Male 11.1 | Intussusception. Resection 6″ of ileum and caecum. | NHL (II) | 0 | NHL | 15 Gy to abdomen | 4.9 | AMML |
| 5. Male 8.4 | Lymphadenopathy and mediastinal mass. Diagnosis by bone marrow and lymph node biopsy. | NHL (IV) | 11.5 | T-Cell | 2 Gy to mediastinum 18 Gy to cranium | 3.0 | AMML |
| 6. Male 7.3 | Mediastinal mass, pleural and pericardial effusions. Diagnosis by bone marrow. | ALL ($232 \times 10^9 l^{-1}$) | 100 | T-Cell | 15 Gy to mediastinum 18 Gy to cranium | 1.5 | AMML |
| 7. Male 12.4 | Intussusception. Resection of caecal mass. Bloom's syndrome. | NHL (III) | 0 | NHL | 15 Gy to abdomen | 3.0 | Pre-leukaemia |
| 8. Male 13.9 | Cervical lymph nodes | NHL (II) | 0 | NHL | — | 3.2 | AML |

of 4.7 years and did not respond to chemotherapy. He was not included in the analysis as he was not registered for the trial.

### Results

The child with Hodgkin's disease but treated with the NHL protocol subsequently developed a myxoid sarcoma. This was of different histological characteristics from the malignant fibrous histiocytoma reported in another series (Suster, 1986). Case 3, with early meningeal disease developed a secondary cerebral astrocytoma and died without further treatment.

Six of the eight patients developed acute myeloid leukaemia or pre-leukaemia, the characteristics of which are given in Table II. The last case developed acute myeloblastic leukaemia but the other five cases all had monocytic or myelomonocytic features. The child described in case 7 had severe neutropenia for several months and marrows taken during this time showed evolution towards M4 myeloid leukaemia. The leukaemic cells in two patients had chromosome abnormalities, including an 11:16 translocation in one.

Further chemotherapy was given to 6 of the 8 children. The girl who developed the undifferentiated sarcoma responded to a combination of ifosfamide, etoposide and *cis*-platinum. Two of the children with acute myelomonocytic leukaemia remain in complete remission. Case 1 was treated on the basis of the BFM protocol (Creutzig *et al.*, 1985) for acute myeloid leukaemia and case 5 has had a successful bone marrow transplant after partial response to cytosine arabinoside and etoposide. He was conditioned with cyclophosphamide and irradiation before grafting from his HLA identical brother.

These 8 patients who developed second malignancy include 7 boys and 1 girl, whereas the trial had a ratio of 3:1 boys. If the girl is excluded as she did not have NHL or T-cell leukaemia then it might be surmised that boys are at special risk of developing secondary disease, especially myeloid leukaemia. The mean age of the children in this report (9 years) does not differ significantly from that in the trial (8 years).

The incidence of second malignancy based on Kaplan–Meier statistical analysis (Kaplan & Meier, 1958), is shown in Figure 2 and shows a risk of 7.8% at seven years. A similar analysis excluding case 8, the boy with Bloom's syndrome (see **Discussion**) gives an incidence of 6.7% at seven years.

### Discussion

This paper describes a relatively high incidence of secondary myeloid leukaemia in a group of children with NHL. A previous study (Mike *et al.*, 1982) included 1,050 children treated for NHL and followed for up to 20 years and did not describe any patients with secondary myeloid leukaemia. A later review by the same late effects study group (Meadows *et al.*, 1985) describes one child with leukaemia among 12 second malignancies in patients surviving NHL. However, all the children in this paediatric series were diagnosed before 1970, and had not therefore received the kind of chemotherapy which has so greatly improved prognosis subsequently (Wollner *et al.*, 1976). There were no cases of myeloid leukaemia in a series of 31 second malignancies among 630 adults treated for NHL (MacDougall *et al.*, 1981).

A recent editorial (Lancet, 1985) however, has commented on the incidence of secondary leukaemia in lymphoma

**Table II** Characteristics of the leukaemias/pre-leukaemia occurring after treatment of NHL or T-ALL

| | *Case 1* | *Case 4* | *Case 5* | *Case 6* | *Case 7* | *Case 8* |
|---|---|---|---|---|---|---|
| *White cell count* × $10^9\,l^{-1}$ | 2.7 | 2.8 | 7.4 | 112 | 1.0 | 2.2 |
| *Marrow blasts* (%) | 85 | 40 | 12 | 90 | 6 | 40 |
| *Morphology* | Large blasts with basophilic cytoplasm | Abnormal myeloid forms | Mixed myeloid and nomocytoid differentiation | Large pleomorphic blast cells | Abnormal myelopoiesis | Abnormal myeloid forms |
| *Histochemistry* | | | | | | |
| PAS | −ve | −ve | −ve | −ve | −ve | −ve |
| Sudan Black | −ve | −ve | −ve | −ve | −ve | +ve |
| Non-specific esterase | +ve | +ve in 30% | Mixed +ve | −ve | +ve in 30% | |
| *T-cell markers* | | | | | | |
| Tdt | −ve | 0% | −ve | | −ve | |
| Pan T, TII | −ve | 39% | −ve | 1% | | |
| *Common ALL* | −ve | | | | | |
| *Myeloid markers* | | | | | | |
| My906, OKM-1 | 90% | 73% | 48% | 60% | | |
| *HLA-DR* | | 94% | | 86% | | |
| Fab classification | M5 | M4 | M4 | M4 | Pre-leukaemia (M4) | M2 |
| *Chromosomes* | 46XY | 47XY+21+e−3 17q−21q+ | 46XY t(11.16) | | | |
| *Treatment* | DR, VP16 CA | VC, Pred ASP, 6MP CA, VP16 vindesine | CP, VP16 allogeneic marrow transplant | VC, Pred ADR CP ASP, TG and CA | None | Mitoxantrone an CA |
| *Response* | Remission >1 year | 2 brief remissions followed by death in relapse | Remission >2 years | Remission 4 months, relapsed and died | Died | Not yet evaluate |

ADR = adriamycin; ASP = asparaginase; CA = cytosine arabinoside; CP = cyclophosphamide; DR = daunorubicin; 6MP = mercaptopuri Pred = prednisolone; TG = thioguanine; VC = vincristine; VP16 = etoposide; AT = azathioprine.

**Figure 2** Time to second malignancy (Figures indicate patients at risk at each event in the study).

patients. Evidence of myeloid leukaemia arising in adult patients treated for both Hodgkin's disease and NHL is described by Pedersen-Bjergaard *et al.* (1985) with the report of 16 NHL patients developing myeloid leukaemia and by Michels *et al.* (1985) who reported 4 such patients. These papers also comment on the incidence of leukaemia after Hodgkin's disease. The earlier review by Grunwald & Rosner (1982) had already collated an extensive series of 216 cases of Hodgkin's disease who developed acute myeloid leukaemia. This paper is especially relevant to our study as it shows a high proportion of myeloblastic leukaemia (45%) similar to the high incidence of M2 leukaemia shown by Michels (1985) in contrast to our results. Also, the paper attempts to show that the majority of their patients had received both alkylating agents and radiotherapy as had our patients.

The association of radiotherapy with second malignancy is well known. One report (Potish *et al.*, 1985) gave a relatively low estimate of 9.6% 30 years after megavoltage irradiation of children but included only one child with NHL.

Six of our eight children had received radiotherapy either as cranial prophylaxis for T-cell disease, as emergency treatment for proptosis or as adjuvant radiotherapy to local disease.

Our estimate of 7.8% at 7 years from a population of 261 children treated with the UKCCSG regimens indicates a significant risk of second malignancies in children surviving NHL after these treatments. The graph has not yet reached a stable plateau and a longer period of observation may show further second malignancies. It is of interest and perhaps predictable that one of the children described had Bloom's syndrome, which is known to predispose to malignancy

(Sawitsky *et al.*, 1966), but even if this child is excluded our estimate gives an incidence of 6.7% second malignancies at 7 years. A comparable figure of 9.9% at 9 years was reported in adult Hodgkin's disease (Pedersen-Bjergaard & Larsen, 1982).

The secondary leukaemias described in our series had chromosome abnormalities documented in two of the three cases studied. Similar findings were reported in the studies of Michels (1985) and Pedersen-Bjergaard *et al.* (1984).

Further trials of treatment for NHL in children should examine the potential role of intensive chemotherapy and radiotherapy as causative agents of secondary malignancy, especially myelomonocytic leukaemia in boys. Adjuvant radiation was discontinued by our Group on the basis of the results of these randomised trials (Mott *et al.*, 1984*a*, *b*). It will be informative to follow the group who received no adjuvant radiation, both in the trial cohort and in the subsequent patients who received a modified chemotherapy regimen.

We acknowledge the assistance of other members of the UKCCSG, particularly Drs M. Radford, J. Martin, I. Hann and Miss J.M. Barnes for statistical advice and the Cancer Research Campaign, the Leukaemia Research Fund and Cancer and Leukaemia in Childhood Trust for financial support.

### References

CREUTZIG, U., RITTER, J., RIEHM, H. & 10 others (1985). Improved treatment results in childhood acute myelogenous leukaemia: A report of the German Co-operative study AML-BFM-78. *Blood*, **65**, 298.

DARBYSHIRE, P.J. & MOTT, M.G. (1986). Secondary acute myeloid leukaemia in a boy with T-cell lymphoma: Successful treatment by bone marrow transplantation. *Clin. Lab. Haematol.*, **8**, 71.

EDITORIAL (1985). Second malignancies in lymphoma patients. *Lancet*, **ii**, 1163.

GRUNWALD, H.W. & ROSNER, F. (1982). Acute myeloid leukaemia following treatment for Hodgkin's disease: A review. *Cancer*, **50**, 676.

HAWORTH, C., STEVENS, R.D.F. & TESTA, N.G. (1985). Serial incidence of bone marrow GM-CFC prior to the development of acute non-lymphoblastic leukaemia in a child treated for non-Hodgkin's lymphoma. *Br. J. Haematol.*, **59**, 79.

KAPLAN, E.L. & MEIER, P. (1958). Non parametric estimation from incomplete observations. *J. Am. Stat. Assoc.*, **53**, 457.

MacDOUGALL, B.K., WEINERMAN, H.B. & KEMEL, S. (1981). Second malignancies in non-Hodgkin's lymphoma. *Cancer*, **48**, 1299.

MEADOWS, A.T., BAUM, E., FOSSATI-BELLANI, F. & 9 others (1985). Malignant neoplasms in children: An update from the Late Effects Study Group. *J. Clin. Oncol.*, **3**, 532.

MICHELS, S.D., McKENNA, R.W., ARTHUR, D.C. & BRUNNING, R.S. (1985). Therapy related AML and myelodysplastic syndrome: A clinical and morphological study of 65 cases. *Blood*, **65**, 1364.

MIKE, V., MEADOWS, A.T. & D'ANGIO, G.J. (1982). Incidence of second malignant neoplasms in children: Results of an international study. *Lancet*, **ii**, 1326.

MOTT, M.G., EDEN, O.B. & PALMER, M.K. (1984*a*). Adjuvant low dose radiation in childhood non-Hodgkin lymphoma. *Br. J. Cancer*, **50**, 463.

MOTT, M.G., CHESSELLS, J.M., WILLOUGHBY, M.L.N. & 4 others (1984*b*). Adjuvant low dose radiation in childhood T-cell leukaemia/lymphoma. *Br. J. Cancer*, **50**, 457.

MURPHY, S.B. & HUSTU, H.O. (1980). A randomised trial of combined modality therapy of childhood non-Hodgkin's lymphoma. *Cancer*, **45**, 630.

PEDERSEN-BJERGAARD, J. & LARSEN, S.O. (1982). Incidence of acute non-lymphocytic leukaemia, pre-leukaemia and acute myeloproliferative syndrome up to 10 years after treatment of Hodgkin's disease. *New Engl. J. Med.*, **307**, 965.

PEDERSEN-BJERGAARD, J., PHILIP, P., PEDERSEN, N.T. & 4 others (1984). Acute non-lymphocytic leukaemia, pre-leukaemia, and acute myeloproliferative syndrome secondary to treatment of other malignant diseases. *Cancer*, **54**, 452.

PEDERSEN-BJERGAARD, J., ERSBØLL, J., SØRENSEN, H.M. & 6 others (1985). Risk of acute non lymphocytic leukaemia and preleukaemia in patients treated with cyclophosphamide for non-Hodgkin lymphomas. *Ann. Int. Med.*, **103**, 195.

POTISH, R.A., DEHNER, L.P., HASELOW, R.E., TAEWAN, H.K., LEVITT, S.H. & NESBIT, M. (1985). The incidence of second neoplasms following megavoltage radiation for paediatric tumours. *Cancer*, **56**, 1534.

ROSE, P.E., AL-RUBEI, K., BEDDALL, A. & HILL, F.G.H. (1985). Acute non-lymphoblastic leukaemia occurring in a boy on treatment for T-cell acute lymphoblastic leukaemia. *Eur. Paediat. Haematol. Oncol.*, **2**, 49.

SUSTER, S. (1986). Transformation of Hodgkin's disease into malignant fibrous histiocytoma. *Cancer*, **57**, 264.

SAWITSKY, A., BLOOM, D. & GERMAN, J. (1966). Chromosome breakage and acute leukaemia in congenital telangiectatic erythema and stunted growth. *Ann. Intern. Med.*, **65**, 487.

WOLLNER, N., BURCHENAL, J.H., LIEBERMEN, P.H. & 3 others (1976). Non-Hodgkin.s lymphoma in children: A comprehensive study of two modalities of therapy. *Cancer*, **37**, 123.